# Giant intrapulmonary solitary fibrous tumor with signs of malignancy

**DOI:** 10.1093/jscr/rjad741

**Published:** 2024-01-16

**Authors:** Kristina Marcinkevičiūtė, Žymantas Jagelavičius, Edvardas Žurauskas, Ričardas Janilionis

**Affiliations:** Faculty of Medicine, Vilnius University, Vilnius LT-03101, Lithuania; Department of Thoracic Surgery, Center of Cardio-Thoracic Surgery, Clinic of Chest Diseases, Immunology and Allergology, Institute of Clinical Medicine, Faculty of Medicine, Vilnius University, Vilnius LT-08661, Lithuania; Department of Pathology and Forensic Medicine, Institute of Biomedical Sciences, Faculty of Medicine, Vilnius University, Vilnius LT-08406, Lithuania; Department of Thoracic Surgery, Center of Cardio-Thoracic Surgery, Clinic of Chest Diseases, Immunology and Allergology, Institute of Clinical Medicine, Faculty of Medicine, Vilnius University, Vilnius LT-08661, Lithuania

**Keywords:** solitary fibrous tumor, intrapulmonary tumor, lobectomy, lung cancer

## Abstract

Solitary fibrous tumor (SFT) is an extremely rare mesenchymal neoplasm usually detected in the pleura, which generally follows a benign course. The localization inside lung parenchyma has more rarely been reported. We present a case of a 51-year-old male with a dry cough, dyspnea, chest pain, and increased perspiration. Radiological images revealed a giant circumscribed mass on the right side of the chest. A transbronchial cryobiopsy of the lung was performed and revealed an SFT. The right upper lobectomy through lateral thoracotomy was performed. The pathological examination confirmed an SFT with a central zone of necrosis that is a sign of malignancy. At a 2-year follow-up, the patient is free of symptoms and with no evidence of recurrence. Although the intrapulmonary localization of an SFT is a rare entity, we should be aware of it as a potential malignant pulmonary neoplasm.

## Introduction

A solitary fibrous tumor (SFT) is an exceedingly rare neoplasm that is generally discovered in the pleura and is classified as a benign mass; however, sometimes it can progress into malignant [1]. The management of the tumor is surgical resection [2, 3]. There are only a few cases of intrapulmonary SFT presented in the literature. No reliable large-sample studies can identify the appropriate diagnostic, therapeutic, and follow-up steps for this tumor. We aimed to present a case of a giant SFT located inside the lung tissue and to report our experience in diagnostics and management.

## Case report

A 51-year-old man was admitted to the hospital because of a dry troublesome cough, pain on the right side of the chest, and increased perspiration. Symptoms lasted for 2 weeks. There had been no previous reports of fever, hemoptysis, chest discomfort, or specific sweats. He had a history of smoking (15 pack-years) and had no data of asbestos exposure.

**Figure 1:**
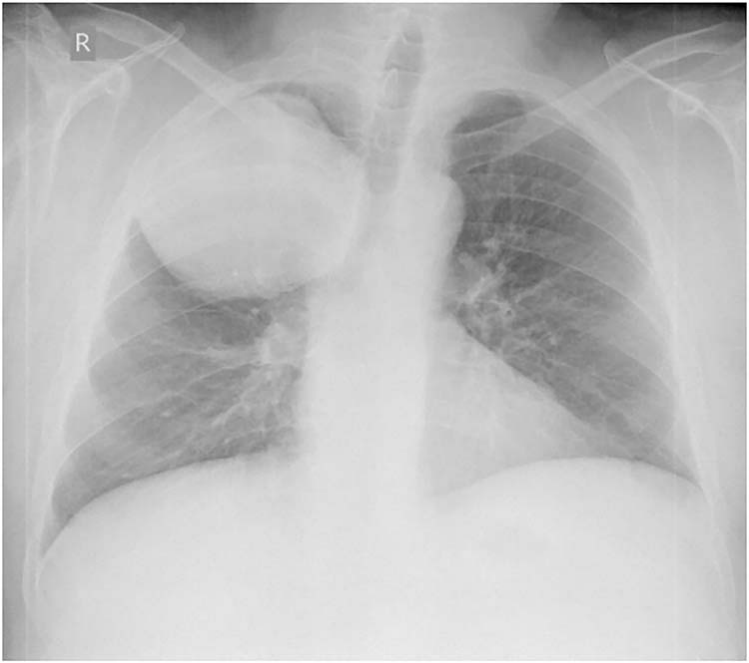
Anteroposterior chest X-ray. Giant, homogeneous, clearly circumscribed opacity in the upper part of the right chest.

Laboratory tests revealed leukocytosis –10.46 (*10^9/l) with 69.2% of neutrophils, but C-reactive protein was normal (1.37 mg/l). Chest X-ray showed a giant, homogeneous, clearly circumscribed opacity in the upper lobe of the right lung (Fig. 1).

**Figure 2:**
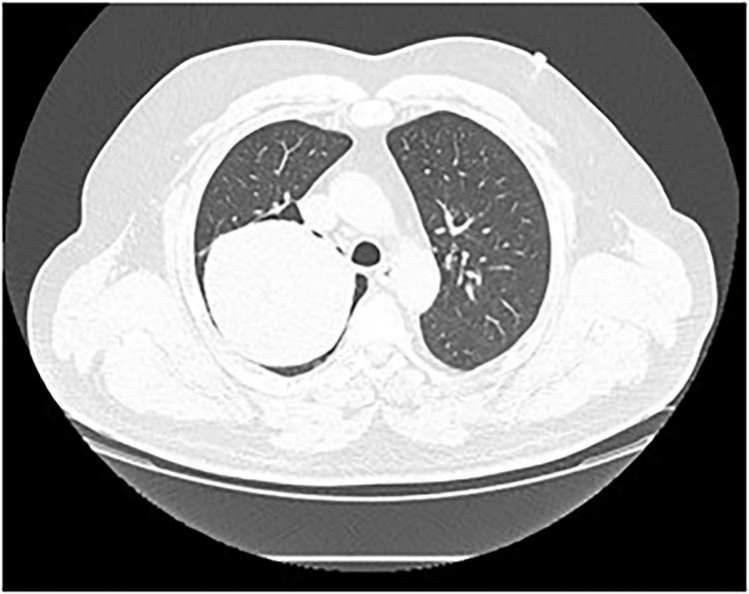
Chest CT scan. A well-defined solid nodule of 10 cm in diameter in the right upper lobe.

Chest computed tomography (CT) revealed a well-defined solid nodule (Fig. 2). Bronchoscopy and transbronchial biopsy were performed. On pathology, no specific findings were found. The bronchoscopy was repeated, and a transbronchial cryobiopsy was performed. On pathology, an abundantly vascularized SFT was verified.

**Figure 3:**
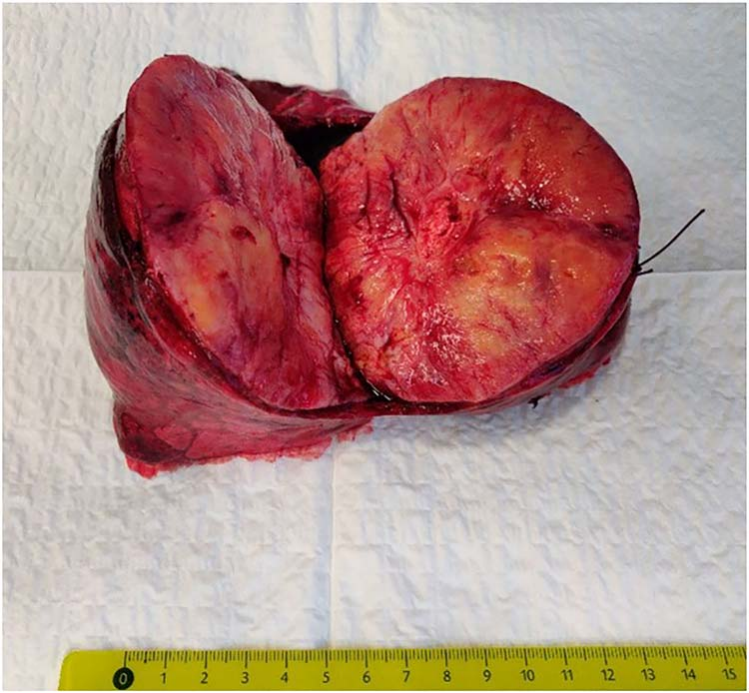
The intrapulmonary tumor.

**Figure 4:**
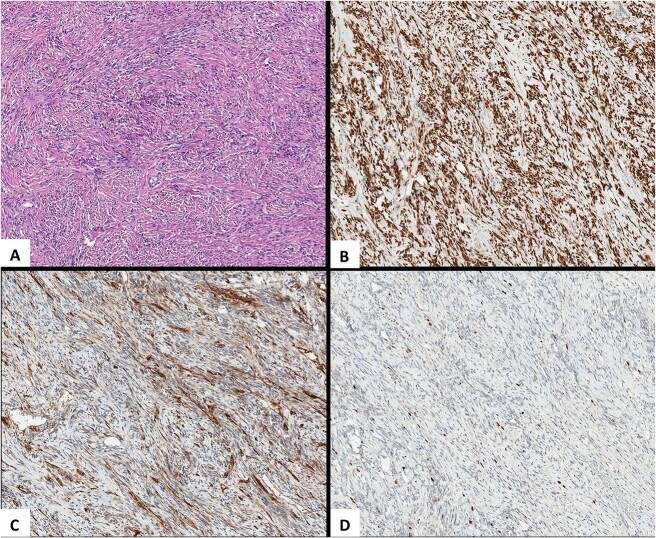
Pathological images. (A) HEx10. An SFT is composed of spindle-shaped cells with indistinct cell borders arranged haphazardly or in short, ill-defined fascicles. (B) STAT6x10. Highly sensitive (98%–100%) and specific (100%) nuclear marker for SFT at all anatomical locations, regardless of tumor morphology. (C) CD34x10. An SFT with limited membranous CD34 expression. (D) Ki-67x10. An SFT, Ki-67 (nuclear marker of cell proliferation) expression.

It was decided to perform pulmonary resection. A right upper lobectomy through lateral thoracotomy was performed, and the giant tumor (size 10 × 7 × 9.5 cm) inside of it was removed (Fig. 3). Macroscopically, the tumor appeared whitish with foci of fibrosis and a necrotic area (4.3 × 2.5 cm) in the center of it. The tumor consists of spindle cells arranged in irregular fibers in a fibrous stroma with thin-walled blood vessels. Focal areas of hypercellularity and necrosis (about 15%) were visible. Mitoses locally were up to 4/10 high-power field. Tumor cells were positive for STAT6 (+++) 100%, CD34 (+/+++) 60%, and Ki67(++/+++) 7% (Fig. 4). The final pathological diagnosis was SFT of the lung with malignancy evidence because of central necrosis, however, without increased focal mitotic activity.

The postoperative period was uneventful. On the 10th postoperative day the patient was discharged.

The follow-up was considered at 1, 6, 12, and 24 months after the discharge. At follow-up, the patient was doing well and was free of symptoms. There was no evidence of recurrence on the chest CT scan.

## Discussion

We have reported a rare case of a giant SFT with signs of malignancy in a nontypical place.

SFTs are composed of spindle-shaped cells and were originally considered neoplasia of the mesothelium because of their propensity to arise from the pleura and mediastinum. In the original description by Klemperer and Coleman in 1931 it was called a “localized mesothelioma” [4]. Since its classic description, SFTs have been described in multiple extraserosal tissues including the lung, liver, thyroid, paranasal sinuses, and orbit, making it less likely to be derived from mesothelium, and more likely to be from a fibroblastic differentiated cell type in the submesothelial mesenchyme [5].

The majority of SFTs are benign; however, patients with malignant SFT present with larger tumors (bigger than 10 cm) and tend to be symptomatic [3, 6]. In the presented case, the symptomatic patient, and the big tumor size (10 cm) suggested a possible malignancy.

SFTs are characterized by haphazardly arranged fibroblast-like cells, indistinct nucleoli, prominent vasculature with perivascular fibrosis, and variable stromal collagen. Microscopically SFT shows a “patternless pattern” with abundant collagen in between cells of the tumor [7].

Although there are no definite criteria for malignancy, the 2013 World Health Organization classification recognizes that hypercellularity, moderate cellular atypia, >4 mitoses per 10 high-power fields, necrosis, nuclear pleomorphism, high MIB-1 staining, and infiltrative margins are histopathologic markers of malignancy and a more aggressive type of SFT [8]. SFTs have a particular pattern of immunohistochemical staining in addition to their normal light microscopic appearance [8]. Tumor cells are positive for vimentin, CD34, CD99, and Bcl2, but SFT is negative for cytokeratin, desmin, alpha-smooth muscle actin, or S100 protein cytokeratin, which are expressed in mesotheliomas. These markers help to differentiate those tumors from others [9, 10]. STAT6 is also a very sensitive and specific immunohistochemical marker for SFT [11]. The expression of Ki67 is strongly associated with malignant tumor cell proliferation and growth [12]. In the reported case, the tumor was positive for STAT6 (+++) 100%, CD34 (+/+++) 60%, and Ki67(++/+++) 7%. Since Ki67 was not expressed, the tumor tended to look benign; however, the clear positive expression of STAT6 and CD34 supported SFT diagnosis.

Surgical management is the gold standard of treatment for intrapulmonary SFTs [2, 3]. The technique of the surgery mainly depends on the size of the tumor. Certain publications propose thoracoscopic surgery for tumors < 5 cm in size, whereas bigger tumors can be removed through a thoracotomy [13]. In the presented case, the tumor was big, so an open lobectomy was carried out.

The prognosis of SFTs depends on pathologic findings. The important prognostic factor associated with overall survival is a free resection margin [2]. In general, the 10-year overall survival rate is up to 94% [14]. Multicenter analysis revealed a significant correlation between metastasis and a larger than 10 cm tumor size with pathological signs of malignancy [15]. In the reported case, central zone of necrosis indicates tumor malignancy and could be related to a higher risk of recurrence. However, during the 2-year follow-up, there was no evidence of recurrence.

We presented an extremely rare case of intrapulmonary SFT with some signs of malignancy. Although SFTs usually have quite a favorable prognosis, some of them might progress locally or metastasize. A careful longer follow-up is required, especially for patients with positive resection margins, larger tumors, and presented malignancy signs.

## Conflict of interest statement

None declared.

## Funding

None declared.
